# Fecal Occult Blood Test Results of the National Colorectal Cancer Screening Program in South Korea (2006–2013)

**DOI:** 10.1038/s41598-017-03134-9

**Published:** 2017-06-05

**Authors:** John Hoon Rim, Taemi Youk, Jung Gu Kang, Byung Kyu Park, Heon Yung Gee, Jeong-Ho Kim, Jongha Yoo

**Affiliations:** 1 Department of Laboratory Medicine, Severance Hospital, Yonsei University College of Medicine, Seoul, Korea; 2 Department of Medicine, Yonsei University Graduate School of Medicine, Seoul, Korea; 3 Department of Pharmacology, Brain Korea 21 PLUS Project for Medical Sciences, Yonsei University College of Medicine, Seoul, Korea; 4 Research Institute, National Health Insurance Service Ilsan Hospital, Goyang, Korea; 5 Department of Statistics, Korea University, Seoul, Korea; 6 Department of Surgery, National Health Insurance Service Ilsan Hospital, Goyang, Korea; 7 Division of Gastroenterology, Department of Internal Medicine, National Health Insurance Service Ilsan Hospital, Goyang, Korea; 8 Department of Laboratory Medicine, National Health Insurance Service Ilsan Hospital, Goyang, Korea

## Abstract

There has been controversy regarding the clinical utility of fecal occult blood test (FOBT) as a screening tool for colorectal cancer (CRC) in the general population. The purpose of this study was to examine the results of Korea national CRC screening using FOBT from 2006 to 2013 and to evaluate the implementation of the program. We analyzed the results of FOBT, colonoscopy, and the side effects during colonoscopy for the subjects (n = 20,609,909) from the Korea National Health Insurance Cancer Screening database. For evaluation of Korea national CRC screening program implementation over the 8-year period, we calculated uptake rate, FOBT positivity rate, and subsequent test compliance rate. The overall uptake rate was 30.1%, with an increasing pattern from 2006 to 2011. A relatively higher FOBT positivity rate (6.4%) and lower subsequent test compliance rate (46.6%) were observed in comparison to the results previously reported in Western countries. Side effects reported within 3 months period after colonoscopy accounted for 0.17% of all procedures, with bleeding being the most prevalent type. Although the implementation of CRC screening program using FOBT in Korea seems successful, trends in key indicators for Korea national CRC screening program should be monitored continuously.

## Introduction

Colorectal cancer (CRC) is the third most common cancer in South Korea after thyroid and gastric cancers^1^. In 2011, the age-standardized incidence rates of CRC were 51.4/100,000 males and 26.4/100,000 females, and the rates continue to increase. When this age-standardized incidence is compared with those of Japan, the United States, and United Kingdom, Korea shows the highest incidence of CRC in both males and females^2^. Even though Korea is considered to be a developed Asian country, the high consumption of red meat or alcohol, and obesity have been suggested as causes of the elevated risk of CRC in Koreans. As a result, the Korean Ministry of Health and Welfare implemented a national cancer screening program for five major cancers (stomach, liver, colorectal, breast, and cervical cancer)^3^. The national CRC screening began in 2004 and required all Korean individuals older than 50 years of age. This program was covered by national health insurance for all participants to undergo fecal occult blood test (FOBT) biannually until 2011 and annually starting in 2012.

Researchers in many countries, including Scotland^4^, Germany^5^, United Kingdom^6^, France^7^, Italy^8^, Canada^9^, Croatia^10^, Spain^11^, and Finland^12^ have published the results of national CRC screening by various screening strategies such as FOBT, colonoscopy, and sigmoidoscopy. Although few reports have studied the results of national CRC screening programs for Asian populations^13, 14^ or the Korean population^15–17^, a relatively small size and short period have been limitations in comparison to reports from Western Caucasian populations.

At the same time, various diagnostic tools were assessed to optimize the most cost-effective screening method. Since FOBT is historically preferred, several randomized controlled trials have demonstrated the efficacy of CRC screening using the FOBT through the illustration of 15–33% reduced mortality rates^18–20^. However, there are recent reports doubting the clinical utility of mortality reduction due to FOBT^21, 22^.

Herein, we report the results of the Korea national CRC screening by FOBT for 2006–2013, which is the largest dataset ever analyzed. We evaluated the implementation of the national CRC screening program in Korea by analyzing key performance indicators. Primary objective was to determine outcome parameters of uptake rate, FOBT positivity rate, and subsequent test compliance rate. Secondary objective was to investigate the rate of side effects during colonoscopy and possibly the presumptive positive predictive value of FOBT. We also determined the diagnostic significance of FOBT for CRC and compared colonoscopy results with those in reports from Western countries.

## Methods

### Study population

The cancer screening program is conducted at the national level by the Korean Ministry of Health and Welfare and targets adults older than 50 years of age among medical aid beneficiaries and national health insurance subscribers. All of the eligible population within the framework of the national CRC screening program receives an invitation letter from the National Health Insurance Corporation (NHIC) at the beginning of each calendar year. All of these examinations within the national screening program were performed free of charge at a clinic or hospital designated as a CRC screening unit by the NHIC. Among the target population from 2006 to 2013, the results of individuals who voluntarily participated in the CRC screening program were included in the study after eliminating erroneous results. Erroneous results were defined as missing values, incorrectly entered numbers, and discrepant data. Demographic information, results of FOBT, results of further evaluation [i.e., colonoscopy or double-contrast barium enema test (DCBE)] if performed, and side effects during colonoscopy were obtained from the Korea National Health Insurance Cancer Screening database.

### Screening protocol

During an 8-year study period, the FOBT was consistently performed as the primary screening method. However, different principles of FOBT including qualitative immunochemical and quantitative guaiac tests were utilized according to availability in visited test centers, because the test centers were scattered throughout the country and changed during the study period (i.e., quantitative: 64.5%, qualitative: 35.5%).

A flow chart of the hierarchical CRC screening protocol is described in Supplementary Fig. 1. For the participants with positive FOBT result, colonoscopy or DCBE was conducted as an additional screening test. When colonoscopy found an abnormal result, a biopsy was performed with the polyp removal during the colonoscopy procedure, if indicated. Although the quality of colonoscopy performance by various doctors could not be realistically standardized, abnormal findings in colonoscopy were classified into three categories of polyp, suspicious cancerous lesion, and definitive cancerous lesion (i.e., cancer). Suspicious cancerous lesions were subjectively judged and recorded, based on the medical interpretation of colonoscopic findings by the performing physicians. The term “suspicious cancer lesions” were categorized in the database to express wide spectrum between definitive polyp and definitive cancer, possibly including inflammatory or high-grade dysplastic lesions that required further biopsy procedure.

### Definitions of evaluation parameters

To evaluate the Korea national CRC screening program implementation during an 8-year period, we calculated three key performance indicators suggested by the previous study^4^.

Uptake rate (UR, %): Number of FOBT participants/Number of eligible individuals for national CRC screening program;

FOBT positivity rate (FPR, %): Number of positive FOBT results/Number of FOBTs performed;

Subsequent test compliance rate (SCR, %): Number of colonoscopy or DCBE procedures performed/Number of positive FOBT results;

For the evaluation of FOBT diagnostic performance, the predictive value of positive tests (PPV) of FOBT for polyp, suspicious cancerous lesion, and cancer were analyzed. Unfortunately, we were unable to calculate the sensitivity and specificity due to lack of a proper control group. Additionally, it is important to remind that the calculated PPV in this study does not meet the strictly ideal PPV values which require histopathological determination by biopsy, due to lack of the appropriate data in the Korea National Health Insurance Cancer Screening database.

PPV: Number of abnormal lesions confirmed by colonoscopy or DCBE/Number of positive FOBT results.

For the evaluation of colonoscopy procedural performance, we analyzed the occurrence of side effects during the colonoscopy procedure. The side effects were classified into four categories of bleeding, perforation, infection, and others.

Rate of side effects associated with colonoscopy (SER, %): Number of side effects reported within 3 months period after the procedure/Number of colonoscopy procedures performed.

For detection of each category for side effects, we used Korean National Health Insurance Service (KNHIS) database to look up for the Korean Classification of Diseases (KCD) codes within 3 months period after colonoscopy. In Korea, all citizens are obligated to enroll in the KNHIS. Since all medical providers are required to submit claims to KNHIS, no health care records are duplicated or omitted using KCD similar to the International Classification of Diseases (ICD) for diagnostic codes. “Bleeding” included KCD codes of hemorrhage and hematoma, whereas “perforation” included those of injury, traumatic perforation, or accidental perforation in all parts of colon. “Infection” included KCD codes of infection following a procedure, while “others” represented those of air embolism, shock resulting from a procedure, and complications of surgical and medical care.

### Statistical analysis

Results of FOBT, colonoscopy or DCBE, and biopsy were analyzed by medical statistics specialists (TY *et al*.). Categorical variables regarding the effects of gender and year on each parameter were compared using the Chi-square test, whereas numerical variables were compared using the t-test. A *P* < 0.05 was considered statistically significant. All statistical analyses were performed using SAS version 9.2 (SAS Institute, Cary, NC, USA), MedCalc version 12.7 (MedCalc Software, Ostend, Belgium), and SPSS version 20.0 for Windows (IBM Corp., Armonk, NY, USA).

### Ethics

This study design was reviewed and approved by the Institutional Review Board of the National Health Insurance Service Ilsan Hospital, Gyeonggi-do, Korea. Written informed consent was waived. Additionally, all experiments and methods in this study were performed in accordance with the Declaration of Helsinki.

## Results

### Characteristics of the study population

After the elimination of erroneous results (n = 2,489), the results of a total of 20,609,909 individuals who participated in the CRC screening program were included in the study (Table 1). Female participants were more than male participants in the study population (i.e. 55.0% vs. 45.0%). Although the youngest age group (50–59 years old) accounted for 45.7% of the total population, no one age group represented more than 50% of the study population. Considering the years of the CRC screening program, the number of FOBT participants increased gradually throughout the study period.

**Table 1:** Characteristics of study population among the Korea national colorectal cancer screening program during 8 years (2006–2013).

	FOBT positive (n)	FOBT negative (n)	Total (n)	Total (%)
Gender				
Male	693,131	8,590,962	9,284,093	45.0
Female	618,978	10,706,838	11,325,816	55.0
Age group				
50–59	561,217	8,865,910	9,427,127	45.7
60–69	456,717	6,734,840	7,191,557	34.9
over 70	294,175	3,697,050	3,991,225	19.4
Year				
2006	90,244	1,074,573	1,164,817	5.7
2007	101,394	1,286,726	1,388,120	6.7
2008	125,602	1,706,382	1,831,984	8.9
2009	143,333	2,137,988	2,281,321	11.1
2010	180,650	2,613,868	2,794,518	13.6
2011	196,055	2,861,220	3,057,275	14.8
2012	227,607	3,655,204	3,882,811	18.8
2013	247,224	3,961,839	4,209,063	20.4
Total	1,312,109	19,297,800	20,609,909	100.0

Abbreviations: FOBT, fecal occult blood test.

### Evaluation of the Korea national CRC screening program implementation

**Figure 1:**
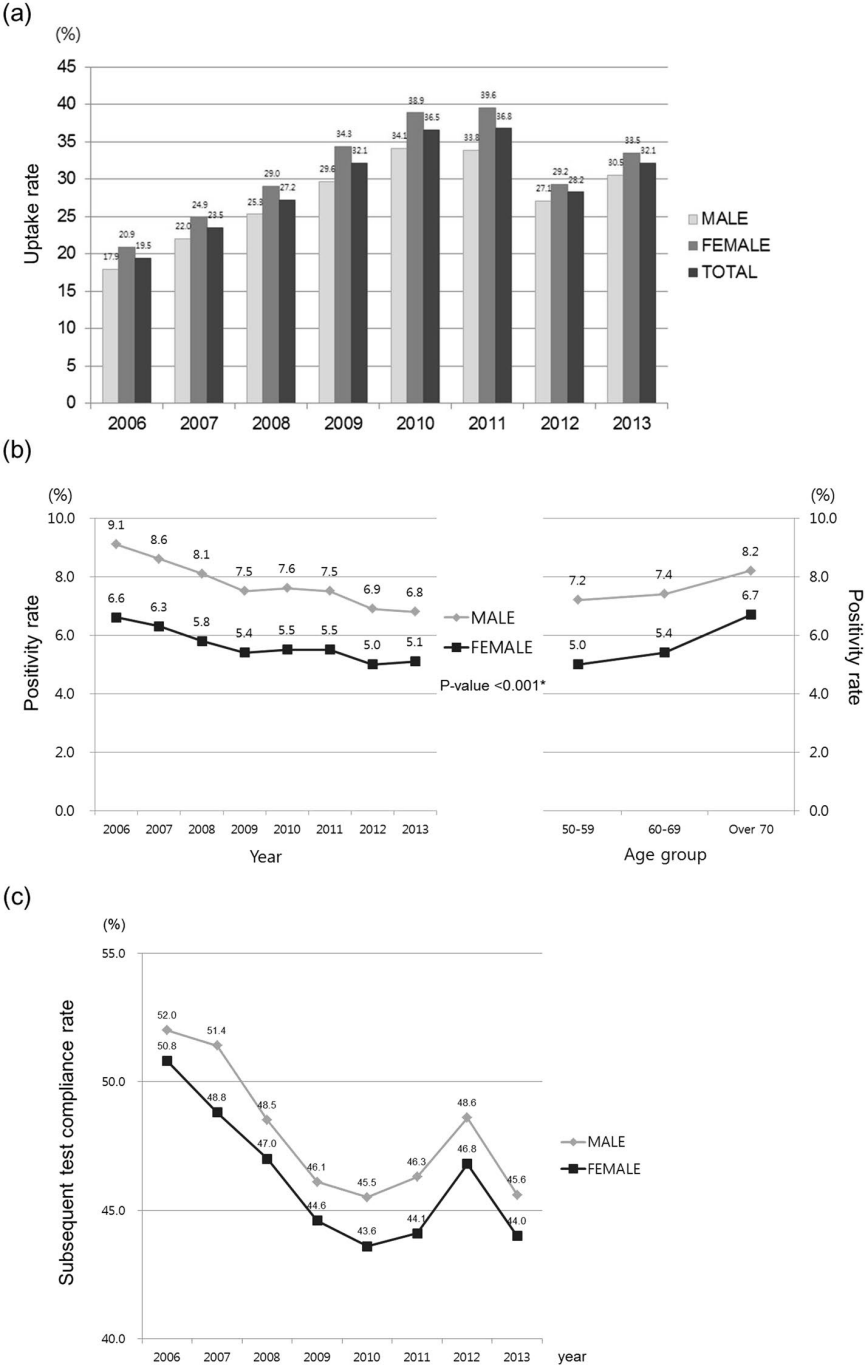
Trends of key indicators in the Korea national colorectal cancer screening program from 2006 to 2013. (**a**) Changes in uptake rates (%) of the fecal occult blood test by gender. Uptake rate (%) was calculated as follows; Number of fecal occult blood test participants/Number of eligible individuals for national colorectal cancer screening program. (**b**) Changes in fecal occult blood test positivity rates (%) by gender and age group. *Comparison of fecal occult blood test positivity rates between males and females and between age groups was performed each year using the Chi-square test. All *P*-values are <0.001. Fecal occult blood test positivity rate (%) was calculated as follows; Number of positive fecal occult blood test results/Number of fecal occult blood tests performed. (**c**) Changes in subsequent test compliance rates by gender. Subsequent test compliance rate (%) was calculated as follows; Number of colonoscopy or double-contrast barium enema test procedures performed/Number of positive fecal occult blood test results.

The changes in UR over the studied years are shown in Fig. 1a. A continuous increase in UR was observed from 2006 to 2011, changing from 19.5% to 36.8%. Due to the time interval change in FOBT uptake from every other year to every year in 2012, the target population increased dramatically in 2012 and 2013 compared to previous years. When this change of national CRC screening program on frequency for FOBT uptake was considered, a similar increase in total UR was observed in 2012–2013, changing from 28.2% to 32.1%. Consistently, the UR of females was higher than that of males in all years.

The total FPR ranged from 5.9% to 7.7% in 2006–2013 and did not show significant trend by year or type of FOBT (quantitative or qualitative). However, FPR was consistently higher in males than females in all years with statistical significance (*P* < 0.001), resulting in higher total mean FPR in males (7.5%) than in females (5.5%) (Fig. 1b). FPR also continuously increased in the older age groups for both genders.

Even though the overall SCR was 46.6%, the SCR of males was higher than that of females in all years (Fig. 1c). Interestingly, there was rising peak of SCR in year 2012 among the decreasing trend in both genders.

### Evaluation of FOBT diagnostic performance

The PPVs of FOBT for polyp, suspicious cancerous lesion, and cancer were 21.4%, 42.6%, and 1.3%, respectively, when abnormal lesions were determined by colonoscopy or DCBE results (Table 2). While PPVs were higher in males than in females for suspicious cancerous lesion and cancer, PPV for polyp was higher in females than in males. When age was considered, PPVs for suspicious cancerous lesion and cancer increased in the older age groups, whereas PPV for polyp decreased. On the contrary, when abnormal lesions were determined by biopsy results, PPVs of FOBT for polyp, suspicious cancerous lesion, and cancer increased to 32.3%, 60.9%, and 6.3%, respectively (data not shown).

**Table 2:** Predictive value of positive test of fecal occult blood test according to colonoscopy or double-contrast barium enema results.

(%)	polyp	suspicious cancerous lesion*	cancer
Total	21.4	42.6	1.3
Gender			
Male	18.2	50.7	1.6
Female	24.8	33.8	1.0
Age group			
50–59	23.4	38.2	0.8
60–69	20.3	44.9	1.5
over 70	18.4	49.0	2.2

*Suspicious cancer lesions were categorized in the database based on individual physicians’ descriptions to express wide spectrum between definitive polyp and definitive cancer, possibly including inflammatory or high-grade dysplastic lesions that required further biopsy procedure.

### Evaluation of colonoscopy procedural performance

A total of 785 side effects were reported within 3 months period after colonoscopy among 473,960 individuals who underwent colonoscopy (i.e., total SER: 0.17%). Among the four categories of side effects, bleeding was the most prevalent side effect in all age groups for both genders (Table 3). When the association between old age and incidence of side effects was analyzed, only perforation showed an increasing pattern in the older age groups with statistical significance (*P* for trend: <0.001 in males and 0.037 in females).

**Table 3:** Events of side effects occurred within 3 months after colonoscopy according to gender and age group.

Gender	Male				Female				Male	Female	*P* value
Age group	50–59	60–69	over 70	*P* for trend	50–59	60–69	over 70	*P* for trend			
Bleeding	107 (0.10)	97 (0.11)	67 (0.15)	0.013	47 (0.04)	54 (0.07)	21 (0.05)	0.156	271 (0.11)	122 (0.05)	<0.001
Perforation	68 (0.06)	86 (0.10)	59 (0.13)	<0.001	30 (0.03)	32 (0.04)	19 (0.05)	0.037	213 (0.09)	81 (0.04)	<0.001
Infection	27 (0.02)	13 (0.01)	11 (0.02)	0.621	13 (0.01)	8 (0.01)	4 (0.01)	0.735	51 (0.02)	25 (0.01)	0.007
Others	5 (0.00)	7 (0.01)	3 (0.01)	0.510	4 (0.00)	2 (0.00)	1 (0.00)	0.674	15 (0.01)	7 (0.00)	0.121

Values are presented in units of number (%). Corresponding Korean Classification of Diseases (KCD) codes that had been input in the Korean National Health Insurance Service (KNHIS) database within 3 months period after colonoscopy for each category are as follows; bleeding: hemorrhage, and hematoma; perforation: injury, traumatic perforation, and accidental perforation; infection: infection following a procedure; others: air embolism, shock resulting from a procedure, and complications of surgical and medical care.

## Discussion

The UR, the most classical representative parameter of the national CRC screening program, was higher in females than males for all years in this study, which is in line with previous reports from nationwide FOBT screening in Scotland^4^, United Kingdom^6^, and France^7^. Considering that there was a higher absolute number of women than men in the study population, a higher UR of FOBT screening in Korean women seems obvious and might be associated with the fact that men are less likely to take advantage of medical services or to seek health advice^23^.

In contrast to UR, FPR and SCR were higher in males than those in females. Although the higher FPR in males can be speculated from the increasing evidence that male gender is a risk factor of CRC^24^, the higher SCR in Korean males than females is an interesting finding. Unfortunately, the plausible underlying reasons for the higher willingness of males to comply with further evaluation in the case of a positive FOBT result were not explained. However, the paradoxical phenomenon of lower UR, higher FPR, and higher SCR in males than females highlights the importance of raising up the UR of males and the necessity of governmental efforts to increase it. For females, we suggest that efforts should be focused on increasing SCR rather than UR. One possible countermeasure to increase UR that can be adapted from a previous study is the use of verbal contact with a health professional or the offer of a telephone consultation for colonoscopy^25^.

When we compare UR, FPR, and SCR results from the Korea national CRC screening program with results of other countries, Koreans showed lower UR, higher FPR, and lower SCR than Western populations (i.e., Scotland^4^, United Kingdom^6^, France^7^, Italy^8^, and Finland^12^) (Supplementary Table 1). Exceptionally, the UR was higher in Korea than in some countries (i.e., Canada^9, 26^, Croatia^10^, and Spain^11^), and FPR was comparable to that of Croatia^10^. Since these three parameters might be affected by cultural atmosphere and national concern for health in the public, it would be more appropriate to compare key indicators of the national CRC program with those from adjacent Asian countries. Due to the relative scarcity of these systematic studies performed in East Asian countries^27^ compared to Western countries, our study might serve as a milestone for assessing racial or ethnic effects on UR, FPR, and SCR in national CRC screening programs of different countries.

At the same time, distinctive findings revealed in this study should be considered in the future directions for domestic improvement of the Korea national CRC screening program. Because UR and SCR play crucial roles in successful implementation of a nationwide CRC screening program, we suggest that various efforts and focused plans to efficiently increase UR and SCR are required. Moreover, changes in UR, FPR, and SCR over time should also be considered. Considering the change from biennial FOBT uptake to annual FOBT uptake in 2012, the increase in UR from 2006 to 2011 and the similar increase from 2012 to 2013 is expected to continue although no special improvements or promotion have been implanted during the study period. The decrease in FPR from 2006 to 2013 might be stabilized with nationwide standardization of FOBT modality. We suggest that the time-dependent analysis of key indicators for evaluation of successful national CRC screening program implementation should be followed into the future.

A recent study by Spanish researchers presents an excellent example of evaluation for key performance indicators in national CRC screening program^28^. A significant rise in the detection rate for high-risk adenoma in Spanish men was highlighted in that study, which highlights the importance of consistent and accurate monitoring for key indicators of the nationwide CRC screening program. Although such approach was not available in our study, further evaluation utilizing several factors suggested in the previous study^28^, including time to colonoscopy and cancer stage classification, should be considered in the future Korean national CRC screening program.

The evaluation of the diagnostic utility of FOBT in the Korea national CRC screening program was another purpose of this study to assess the successful implementation of the program. Although both quantitative and qualitative methods were utilized in the screening program, the PPVs for suspicious cancerous lesion and cancer were all higher in males than females, which is in line with previous results^23^ and with the evidence of the higher CRC incidence in men^24^. Compared to PPVs analyzed in other countries, PPVs for cancer in Korea appear to be lower, even when compared with values using biopsy results. Nevertheless, PPVs for polyp and suspicious cancerous lesion were higher than those calculated in other countries. Considering the ultimate objective of the screening step, comparable PPVs calculated in this study demonstrate that FOBT performed in the Korea national CRC screening program from 2006 to 2013 showed appropriate diagnostic specification. However, a more careful follow-up study is required since different modalities of FOBT (i.e., guaiac-based or immunochemical FOBT) can affect the PPVs in different CRC screening programs^29, 30^. When financial resources and environmental availability are taken into account, we suggest that a uniformly standardized FOBT modality should be introduced in the future CRC screening in Korea.

One important result of this study that is unique compared to results from other population-based CRC screening reports is the SER analysis in subsequent colonoscopy performed for individuals with positive FOBT results. Since colonoscopy is a confirmative test, explanation on the side effects of the procedure should be offered to participants in advance. Although the quality of performing physicians varies enormously depending on country and district, the potentially life-threatening risks of colonoscopy such as perforation and bleeding must be evaluated in order to apply this procedure to the national population. In terms of overall morbidity, the total SER in this study (0.17%) was lower than those of United Kingdom (0.44%)^31^, Germany (0.28%)^5^, Australia (0.3%)^32^, and France (0.23%)^7^. This finding indicates that the safety and proficiency of colonoscopy in the current Korea national CRC screening program are equivalent to or even better than those of other countries. Our results indicate that males are more prone to the side effects of colonoscopy than females, irrespective of complication type. Another interesting finding regarding SER in this study is that the side effects of perforation and bleeding occurred more frequently in the older age groups. In contrast, infection related to colonoscopy did not show any specific association with age or gender. Based on these results, we suggest that more caution to prevent perforation or bleeding should be exerted by performing physicians, especially in the evaluation of old male participants in the Korea national CRC screening program. Lastly, this study represents the most updated information for Korean population encompassing the period when change of required interval for follow-up screening test occurred, whereas other previous studies limited the study period to 5 years^17, 33^. Focusing on gender-specific trends over 8 years, our study highlights the importance of gender-specific approach in order to improve the key indicators in the national CRC screening program efficiently.

There are several limitations of this study. Firstly, the heterogeneity of the FOBT modalities utilized in this study could have affected the analysis. Although a total proportion of quantitative and qualitative test in the total population was available, the precise modalities of each individual were not collected in the original database unfortunately, due to various environments of clinical laboratories at multiple centers located throughout Korea. However, the interpretation of positive or negative results was reported according to the corresponding modalities. Secondly, the performance of colonoscopy or DCBE could not be standardized because the test centers were also scattered throughout the country. Thirdly, as mentioned before, the histopathological assessment was missing in the database which precluded us from investigating the true PPV values based on pathologic results. However, it would still be valuable to estimate at least the maximum PPV of FOBT since the true PPVs are obviously lower than our calculated PPVs based on colonoscopy results. Fourthly, characteristics of abnormal lesions detected in colonoscopy such as size and location could not be thoroughly investigated for association with FOBT results due to incomplete data in the screening program. Furthermore, data collection for adverse events via classification tools might have underestimated SER due to subjective reporting by individual clinicians. Although the major complications have been elucidated in this study, cautious interpretation for the absolute SER values is required especially when comparing these numbers with other datasets. Finally, socioeconomic deprivation status of participants, which has recently been suggested to be related with low UR and low SCR^34^, was not analyzed in the present study due to the lack of data.

In conclusion, this study presents the largest dataset from a nationwide CRC screening program. Since degrees of participation and subsequent test compliance were as low as other countries, there appear to be several points for improvement in the future amendments of national CRC screening, such as special efforts to increase the UR in males. Trends of key indicators for Korea national CRC screening program should be monitored continuously, in accordance with the Korean-specific characteristics of the CRC and the national screening program itself, which were revealed in this study.

## Electronic supplementary material

Supplementary Table 1 and Supplementary Figure 1
