# Supplementary material for: Fecal Occult Blood Test Results of the National Colorectal Cancer Screening Program in South Korea (2006–2013)
Source: Sci Rep. 2017 Jun 5;7:2804. doi: 10.1038/s41598-017-03134-9 (PMC5459855; doi:10.1038/s41598-017-03134-9)

Scientific Reports: Original Article

Fecal Occult Blood Test Results of the National Colorectal Cancer Screening Program in South Korea (2006-2013)

John Hoon Rim[1,2,3], MD; Taemi Youk[4,5], MS; Jung Gu Kang[6], MD, PhD; Byung Kyu Park[7], MD, PhD; Heon Yung Gee[2,3], MD, PhD; Jeong-Ho Kim[1], MD, PhD; Jongha Yoo[1,8], MD, PhD

[1]Department of Laboratory Medicine, Severance Hospital, Yonsei University College of Medicine, Seoul, Korea

[2]Department of Medicine, Yonsei University Graduate School of Medicine, Seoul, Korea

[3]Department of Pharmacology, Brain Korea 21 PLUS Project for Medical Sciences, Yonsei University College of Medicine, Seoul, Korea

[4]Research Institute, National Health Insurance Service Ilsan Hospital, Goyang, Korea

[5]Department of Statistics, Korea University, Seoul, Korea

[6]Department of Surgery, National Health Insurance Service Ilsan Hospital, Goyang, Korea

[7]Division of Gastroenterology, Department of Internal Medicine, National Health Insurance Service Ilsan Hospital, Goyang, Korea

[8]Department of Laboratory Medicine, National Health Insurance Service Ilsan Hospital, Goyang, Korea

C**orresponding author:** Jongha Yoo, M.D., Ph.D.

Department of Laboratory Medicine,

National Health Insurance Service Ilsan Hospital,

100 Ilsan-ro, Ilsandong-gu, Goyang 10444, Korea

Tel: +82-31-900-0909, Fax: +82-31-900-0925

E-mail: jhyooken@gmail.com

Supplementary Table 1. Comparison of uptake rates, fecal occult blood test positivity rates, and subsequent test compliance rates reported in national colorectal cancer screening for various countries

Country	Scotland	United Kingdom	France	Italy	Canada	Croatia	Spain	Finland	Korea
Reference No.	4	6	7	8	9, 26	10	11	12	Present study
Study period	2000-2002, 2005-2007	2006-2008	2008–2009	2005-2009	2009-2011	2007-2011	2000-2004	2004-2007	2006-2013
UR	55	51.6	34.3	49.6 (1st round)/ 54.4(2nd round)	7.6-14.8	19.9	17.2(1st round)/ 22.3(2nd round)	68(male)/ 80(female)	30.1
FPR	2.07	2.1	2.8	6.2 (1st round)/ 5.9(2nd round)	4.4	6.9	3.4(1st round)/ 0.8(2nd round)	3.1(male)/ 1.6(female)	6.4

SCR	85.5	87.2	88	92.0 (1st round)/ 93.0(2nd round)	80.5	68.5	89.3	90	46.6

Abbreviations: UR, uptake rate; FPR, fecal occult blood test positivity rate; SCR, subsequent test compliance rate.

Supplementary Figure 1. Flow chart of the hierarchical colorectal cancer screening protocol utilized in the Korea national colorectal cancer screening program.

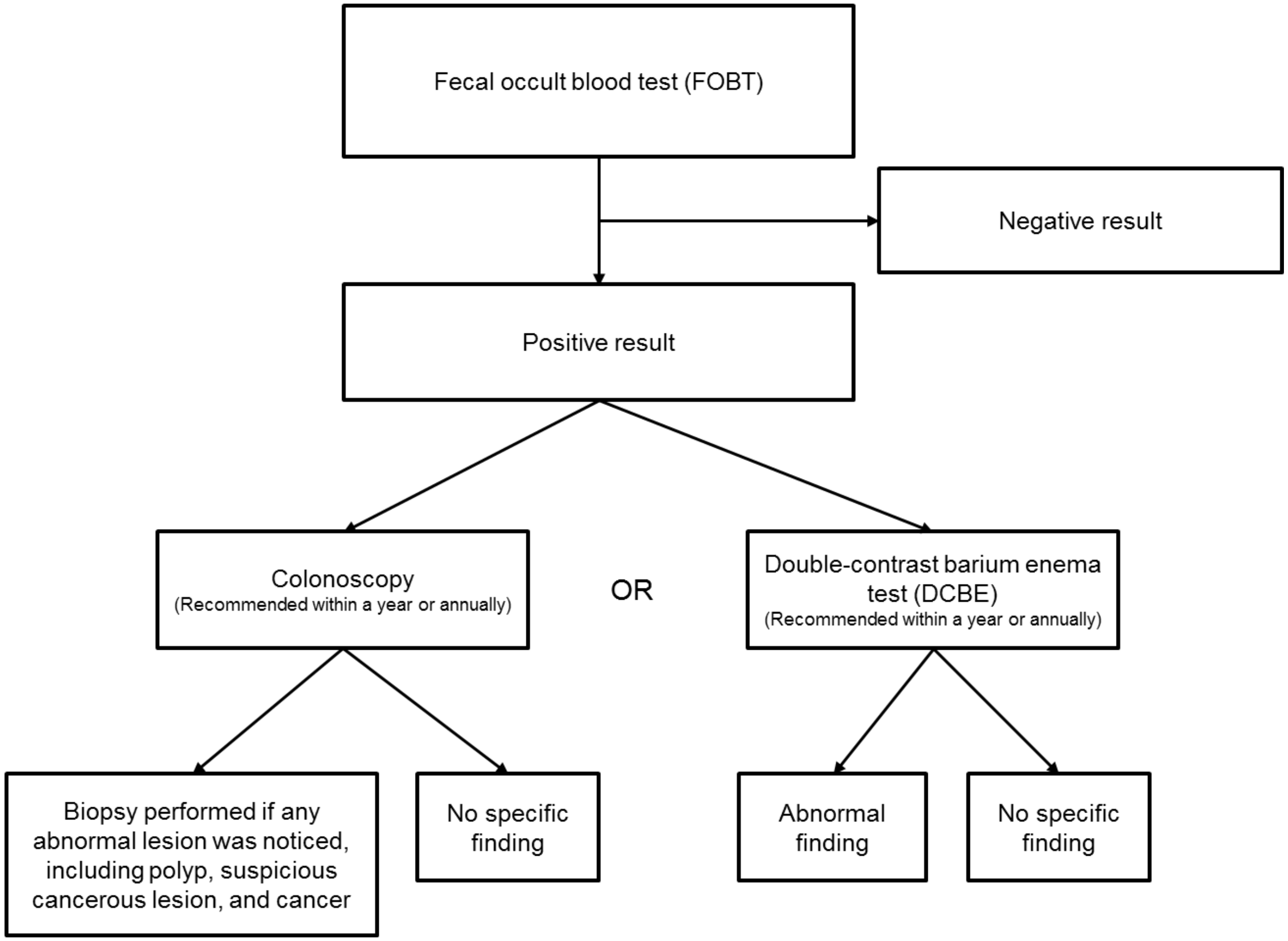

Supplement: Supplementary file 1 — Supplementary Table 1 and Supplementary Figure 1 [file 41598_2017_3134_MOESM1_ESM.pdf]
